# Interactions Between Epilepsy and Plasticity

**DOI:** 10.3390/ph11010017

**Published:** 2018-02-07

**Authors:** José J. Jarero-Basulto, Yadira Gasca-Martínez, Martha C. Rivera-Cervantes, Mónica E. Ureña-Guerrero, Alfredo I. Feria-Velasco, Carlos Beas-Zarate

**Affiliations:** 1 Cellular Neurobiology Laboratory, Cell and Molecular Biology Department, CUCBA, University of Guadalajara, 45220 Zapopan, Jalisco, Mexico; gasca.mx@hotmail.com (Y.G.-M.); mrivera@academicos.udg.mx (M.C.R.-C.); alfredoferia1340@hotmail.com (A.I.F.-V.); 2 Neurotransmission Biology Laboratory, Cell and Molecular Biology Department, CUCBA, University of Guadalajara, 45220 Zapopan, Jalisco, Mexico; murena@cucba.udg.mx; 3 Development and Neural Regeneration Laboratory, Cell and Molecular Biology Department, CUCBA, University of Guadalajara, 45220 Zapopan, Jalisco, Mexico; carlos.beas@academicos.udg.mx

**Keywords:** seizures, hippocampus, granular cells, plasticity, epilepsy

## Abstract

Undoubtedly, one of the most interesting topics in the field of neuroscience is the ability of the central nervous system to respond to different stimuli (normal or pathological) by modifying its structure and function, either transiently or permanently, by generating neural cells and new connections in a process known as neuroplasticity. According to the large amount of evidence reported in the literature, many stimuli, such as environmental pressures, changes in the internal dynamic steady state of the organism and even injuries or illnesses (e.g., epilepsy) may induce neuroplasticity. Epilepsy and neuroplasticity seem to be closely related, as the two processes could positively affect one another. Thus, in this review, we analysed some neuroplastic changes triggered in the hippocampus in response to seizure-induced neuronal damage and how these changes could lead to the establishment of temporal lobe epilepsy, the most common type of focal human epilepsy.

## 1. Introduction

The human central nervous system (CNS) is composed of multiple neuronal communication networks, which are closely regulated by their interactions with non-neuronal cells (glial and endothelial cells) [1]. One of the multiple attributes of the CNS is the ability to restructure itself in response to both physiological and pathological stimuli, through a process known as neuroplasticity, which is determined by cell and molecular mechanisms that modify the structure, density, and functionality of synaptic connections. In general, neuroplastic changes include the following: (a) increments in the efficacy of synaptic transmission in pre-existing synapses; (b) induction of new synaptic connections and reordering of pre-existing contacts; and (c) improvement of the ability of neurons to become excited. These changes were considered for a long time as an exclusive event of early developmental postnatal stages that are mainly stimulated as a subjacent process to learning and memory [2], an ability that was supposed to disappear with the ageing [3,4]. However, after several investigations, it was demonstrated that neuroplasticity is as follows: (1) a continuous process of remodelling neuronal circuitries; (2) it can occur at any life stage in response to different stimuli including brain damage; and (3) it comprises short-, medium- and long-time events that could last from minutes to years. In this sense, it must be considered that even the adult brain conserves the ability to generate neuroplastic responses, and they are lesser than those observed in early developmental stages [5]. In addition, in the adulthood, this process usually emerges as an adaptive and compensatory response to cerebral damage, among other processes [6,7]. Therefore, identifying the mechanisms implied in neuroplasticity is critically needed to improve our understanding of several physiological and pathological processes that occur in the CNS.

Additionally, it must be noted that even if neuroplastic changes try to compensate for the damage, in some cases, they can lead to the establishment of chronic neurological disorders or neurodegenerative illnesses [8,9]. For example, abnormal structural modifications to the dendritic spines have been implicated in intellectual disabilities and in childhood epilepsy [10], while insufficient or excessive elimination of synaptic contacts has been described as a basic aetiological mechanism of certain behavioural disorders, such as schizophrenia initiated at adolescence [11,12].

Epilepsy is a chronic disorder of the CNS characterized by the appearance of spontaneous recurrent seizures (SRS) generated by an imbalance of excitatory and inhibitory synaptic transmissions that induce electrical activity that is abnormally synchronized, which initially is focal, but may generalize [13,14,15,16]. It is still unknown whether this imbalance is a cause or a consequence of this disease. For some types of epilepsy categorized as idiopathic, the etiology is unknown, but it is thought that they may involve genetic predispositions [17,18]. In other cases, epilepsy is categorized as secondary or acquired because the seizures are the result of another neurological disease or an acute brain injury.

After the detonating neuronal damage, several neuroplastic changes predispose the brain to develop SRS in a process known as epileptogenesis, which leads to the establishment of epilepsy [15,16,19,20,21,22]. This process results from progressive cell and molecular changes that lead to neuronal network reorganization; most of these changes occur during a latent period of several years in humans and from weeks to months in experimental models. Because plastic responses of the CNS seem to depend on both the developmental state and the regional susceptibility, not all subjects with brain injuries develop epilepsy [23].

The hippocampus has been clearly identified as an epileptogenic brain region that is highly susceptible to damage, that involves both structural and functional changes, such as neuronal loss, inflammation, blood-brain barrier (BBB) leakage, angiogenesis, neurogenesis, axonal sprouting and synaptogenesis, among others. All these events have been associated with the pathophysiology of the temporal lobe epilepsy (TLE), which remains the most severe and frequent type of pharmacoresistant focally acquired epilepsy [15].

Despite the many physiological implications of neuroplasticity, the aim of this review is to analyse some of the neuroplastic changes triggered in the hippocampus as a response to cell death generated by seizures and how those could lead to the establishment of TLE.

## 2. Neuroplasticity in the Epileptogenic Hippocampus

**Figure 1:**
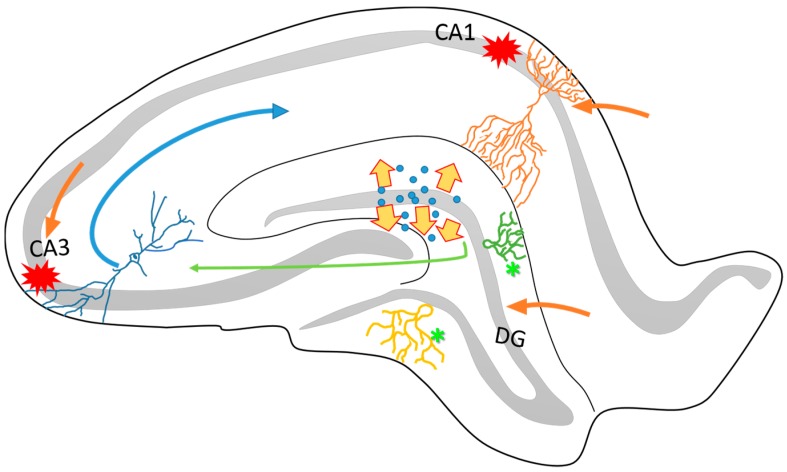
Schematic representation of the structural organization of the rat hippocampus. It is known that the hippocampus is connected to the entorhinal cortex through different anatomical circuitries that have been well described. Particularly, the perforant pathway projects from the DG and the CA3 to the CA1 (green and blue arrows). One of the characteristics of this circuitry is its directionality between the different neuronal layers. The DG, CA3 and the apical layers of CA1 (orange arrows) project mainly via the superficial layers of the entorhinal cortex (II and III). On the other hand, many pieces of evidence have reported that the hippocampus is highly vulnerable to cell loss via seizure activity, particularly in the CA1 and CA3 subfields (red marks). The dispersion of dentate granular cells (yellow arrows) and intense axonal sprouting (asterisks) are common in epileptogenesis process. These structural changes affects the organization and function of hippocampal circuitry and contribute to the establishment of the TLE.

The hippocampus is a cortical structure whose anatomy and plasticity have been broadly studied. It is a prominent C-shaped, bulging structure that is localized in the floor of the temporal horn of the lateral ventricle, which is subdivided into three major subfields (CA1–CA3) in rats and into four (CA1–CA4) in humans. Along with the dentate gyrus (DG), the subicular complex, and the entorhinal cortex comprise the hippocampal formation (commonly referred to as the hippocampus) [24,25]. The principal neurons of the hippocampus (pyramidal and granular cells) are excitatory and are surrounded by several types of interneurons (mainly GABAergic) and aminergic axon terminals. However, the major hippocampal circuitry (trisynaptic; Figure 1) is essentially excitatory and is highly sensitive to synaptic remodelling [26,27]. This brain region has a functional relevance in memory and learning processes, motor control and stereotyped behaviours, among others [28]. The highly organized laminar structure of the hippocampus has permitted the clear identification of cellular changes associated with both neuroplastic and epileptogenesis processes [24]. Moreover, as has been mentioned above, the hippocampus is one of the most vulnerable cerebral regions to be damaged, being fundamental in the establishment of the different types of epilepsy [29,30].

Nearly fifty percent of acquired epilepsies belong to the focal type [31,32], and inside this category, TLE is considered the most frequent type in adults [33,34,35,36,37,38]. Although the exact cause of TLE is unknown, in most cases, it appears after an initial precipitating injury, such as *status epilepticus* (SE), tumours, vascular malformations, traumatic brain injury, severe infections, inflammation, among others cases [15,16,20,21,22]. In TLE, the epileptic focus involves limbic structures, such as the hippocampus, entorhinal cortex and amygdala, although less frequent damage is found in the latter two brain regions [39]. Generally, hippocampal sclerosis (HS) or mesial temporal sclerosis can be present [40], which has been found to be related to massive neuronal loss and reactive gliosis in the mid-basal areas that comprise the temporal lobe [41,42,43]. It is important to consider that, depending on the initial precipitating injury, the HS can be present or absent in the TLE [35,44].

Other changes observed in the hippocampus after epileptic seizures include selective neuronal loss (particularly in the pyramidal layer of CA1 and CA3 subfields, DG and the entorhinal cortex) [41,45,46] and cellular dispersion in the DG [47,48,49,50,51,52], as well as axonal sprouting in granular cells (Figure 1) [53,54,55,56]. These events have been observed in different experimental animal models for the study of epilepsy [46,57,58], as well as in human brain samples obtained from patients with drug-resistant TLE, who have undergone surgery to remove the epileptogenic zone, which is a common strategy that is applied as a treatment in this type of epilepsy [59,60,61].

It should be mentioned that, even though in some cases the seizures are brief, they may be sufficient to produce alterations in cerebral homeostasis and synaptic functioning and to promote new synaptic connections and aberrant circuitries [62]. Different research groups around the world have proposed that neural networks affected by epileptic episodes suffer neuroplastic modifications that contribute to the adoption of different pathological phenotypes [63,64]. However, this statement requires of more studies to clarify the mechanisms implicated in the structural and functional rearrangement of neural networks in epilepsy [65].

Taking into account several results that were obtained through different methodological strategies, it has been postulated that the neuronal loss produced in the hippocampus by epileptic seizures triggers an intense axonal sprouting in the neighbouring granular cells of the DG [58,61], increasing the number of aberrant synaptic connections that, together with an evident decrement in chandelier hippocampal cells (GABAergic interneurons), which exert a significant inhibitory effect [59], contribute to the establishment of hyperexcitable circuitry that stimulates the excessive release of glutamate (Glu), promoting the epileptic activity and excitotoxic neuronal damage [62].

Although, following the damage generated by seizures, several compensatory changes try to maintain the neuronal homeostasis and to restore the neuronal connections, they are limited by the underlying mechanism of epileptogenesis. In some cases of epilepsy (usually pharmacoresistant type), seizures can become so frequent and intense that repair mechanisms are unable to carry out their function [66]. However, the neuroplastic process remains active without restoring normal function and, on the contrary, tends to facilitate the modifications that promote the epileptogenic process.

## 3. Axonal Sprouting: Hippocampal Cell Response to Epileptic Seizures

The neuroplastic mechanisms involved in the recovery from lesions or illnesses are not always positive; and in some cases, they are responsible for initiating or enhancing the pathological processes [62]. When a massive loss of neuronal cells occurs, generating deafferentiation of a brain area, axonal sprouting arises as a widespread plastic response in part of the CNS to reorganize itself and try to restore the damage [67]. Nevertheless, the axonal sprouting process has also been described as a sign of different disorders of the CNS in which neuronal death occurs. Particularly in TLE, the prominent neuronal death produced by the seizures in the hippocampus (CA1 and CA3 subfields) and the amygdala, promotes the sprouting of new axons in the surviving dentate granular cells, which attempt to reinnervate the affected brain area, and produces an aberrant synaptic reorganization, which has been implicated in the pathogenesis of this disease [68,69]. In a clinical study, Scheimeiser and collaborators (2017) analysed 319 samples of TLE patients and observed that there was a correlation between the extent of mossy fibre sprouting and neuronal loss [70], but other evidence has suggested that neuronal degeneration is not strictly necessary for the sprouting to begin [71]. On the other hand, the aberrant function of axonal sprouting has also been considered as a consequence of granular cell ageing. Althaus and colleagues (2017) injected retroviruses carrying a synaptophysin-yellow fluorescent protein in a model of TLE in rats (SE induced by pilocarpine administration) and demonstrated that, in both neonatal and adult animals, the newly born granular cells contributed to the aberrant axonal reorganization to a similar extent, at least in this experimental model [72]. These and other results suggest that there is a more complex relationship between granular cells age and their participation in seizure-related plasticity.

The synaptic reorganization of the CNS and neuroanatomical description of the axonal sprouting process involves a very complicated series of events that are difficult to be reproduced in vitro; however, some of them have been characterized in samples of human brains and animal models of TLE [60,73]. Timm’s staining method [74,75] has evidenced important structural changes in dentate granular cells with the sprouting of new axonal collaterals [54,76] that establish functional synapses with the dendrites of granular cells inside the inner molecular layer [45,74,77]. The neuronal reorganization of networks may occur in different brain areas generating numerous aberrant connections that promote TLE [46,76].

On the other hand, axonal sprouting is a mechanism regulated by different molecules that play an important role in brain development and in epileptogenesis. In this sense, it is known that the expression of some proteins, such as MAPs (microtubule-associated proteins) [62] and the GAP-43, are essential for this process. In particular, the GAP-43 protein, which is abundant in the neuronal growth cones and is required for growth and restructuration of neuronal axons, it is widely utilized as a specific marker of axonal sprouting [78,79,80,81,82,83]. Other critical molecules capable of promoting or inhibiting axonal growth are found outside of the cell, such as extracellular matrix molecules and cell adhesion molecules, as well as diffusible molecules such as cytokines produced by glial (reactive) or neuronal cells around the injured region [83]. These molecules also have other important functions, pointing the way that a new axon has to follow to reach to the target regions and cells.

Despite the different approaches that have so far contributed to the understanding of neuroplasticity and epileptogenesis processes, the identification of more participating molecules that are involved and a detailed description of the neuroanatomical profile of axonal sprouting at the level of individual cells are necessary to improve the treatment of this and other neuronal diseases.

## 4. Transcriptional Changes Related to Seizures and the Neuroplasticity Process

During development or during a pathological event, the neuroplastic process is highly influenced by extrinsic environmental experiences. Through different studies, it has been shown that short- and long-term synaptic plasticity responses may change substantially in the hippocampus and cerebral cortex after epileptic seizures [84,85,86]. However, the underlying mechanisms of these changes are still generally unclear because of contradictory results that currently exist. Immediate early genes (IEGs) are among the first changes induced in response to different physiological or pathological events. Specifically, changes in the gene expression levels of c-Fos, FosB, c-Jun, Egr1, Egr2, Egr4, FoxP2, Homer-1, Nacc-1, Nurr77, Arc and ApoE among others have been observed; these expression changes have been implicated in both the neuroplastic process and in the establishment of neurological disorders, such as epilepsy [87,88,89,90,91,92,93,94,95,96,97,98]. Subsequently, it has been proposed that IEGs are activated by the excessive synaptic activation that is generated in hippocampal and neocortical tissues [97,99,100] by high levels of Glu released to the extracellular space [101]. Glu overactivates its specific receptors, promoting excessive neuronal excitation and the overload of cytosolic free-Ca^2+^, followed by cell death via excitotoxicity [102,103]. Glu-mediated signalling activates kinase cascades, such as the ERK pathway [104,105], that are responsible for phosphorylating several transcription factors that may translocate to the cell nucleus and regulate gene transcription processes [106].

On the other hand, previous studies have documented a complex pattern of long-term changes in plasticity-associated protein expression after seizure activity [107]. Neurotrophins, brain-derived neurotrophic factor (BDNF), insulin-like growth factor (IGF), and vascular endothelial growth factor (VEGF), are just some of the affected proteins [108,109]. In some cases, the low protein expression may be confused with the ageing process, which is known to alter the time course of gene expression, similar to that after seizures activity [110,111]. Additionally, differential changes in the expression of neurotransmitter receptors and modifications to the expression levels of neuropeptides in hippocampal cells, are induced by seizures [112,113,114,115,116,117], such as neuropeptide Y (NPY) in DG cells (Y1, Y2, and Y5), which has been found to be related to memory, learning and epilepsy.

Although we do not know enough of the specific genes that are involved in the neuroplasticity associated with epilepsy, it has been considered that each stimulus may initiate its own molecular pathway activation in the brain depending on the damage intensity generated to the neural networks [118]. The results of studies regarding changes in the expression of genes and the modifications of proteins in a temporal profile to determine its variability could have important implications for the development of new treatments for seizures disorders.

## 5. Changes in the Neurotransmission Systems by Seizures Related to Neuroplasticity

Neurotransmission systems are implied in neuroplasticity as both inducers and as targets of the process. In this sense, although more than fifty transmitter substances have been described, two of them appear to be particularly relevant in all neurological processes, including neuroplasticity and epileptogenesis, namely, Glu and γ-aminobutyric acid (GABA). Both are highly concentrated amino acids that converge biochemically and functionally in most of the regions of the vertebrate CNS; they exerting opposite effects, at least in the adulthood, wherein Glu normally depolarizes and excites neurons, while GABA hyperpolarizes and inhibits them [119,120,121]. In general, it is accepted that principal cortical and hippocampal projection neurons (pyramidal and granular cells) release Glu as a primary neurotransmitter, and most of the surrounding interneurons release GABA, among other neurotransmitters [119,122,123]. Glu-mediated excitation is essential for the neural activation implied in basically all nervous functions [120,121], while GABA-mediated inhibition is involved in excitation threshold maintenance and in the control of neuronal firing frequency and occurrence [14,122,124]. Subsequently, in general, the cross-talk between glutamatergic and GABAergic synapses builds, defines and remodels the neuronal circuitries [14,125] influenced by other neurotransmitters (such as acetylcholine, serotonin and dopamine, among others) and also, by the astrocytes activity because they do not only reuptake and metabolize these neurotransmitters, but they respond specifically to GABA and release Glu [126].

**Figure 2:**
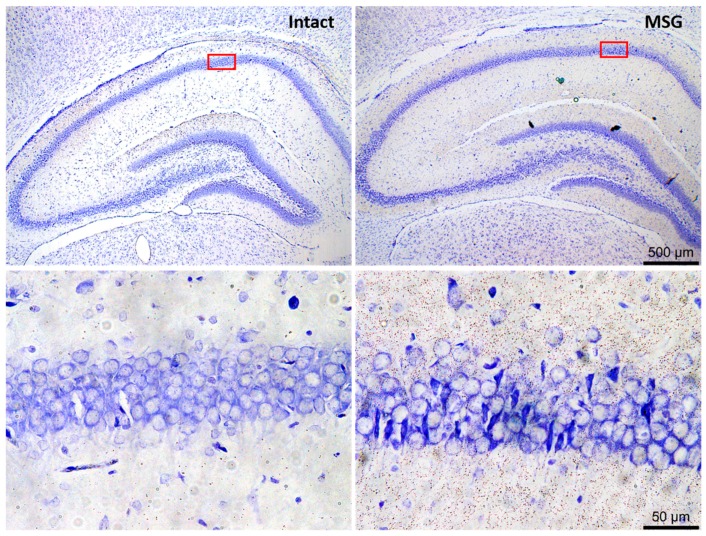
Representative images of neuronal excitotoxic damage in rat hippocampus after subcutaneous monosodium glutamate neonatally administered. Photomicrographs were taken at the level of the dorsal hippocampus, with a focus on the CA1 area (square red). Nissl stain. Scale bars correspond to 500 and 50 µm in upper and lower panels, respectively (for methodological details see: Rivera-Cervantes [131]).

It has been broadly demonstrated that extracellular Glu levels are significantly increased in precipitant conditions of neuronal damage and during the seizures [122,127,128] that Glu-mediated excessive neuronal excitation leads to neuronal damage through a process known as excitotoxicity, which has been widely resembled in the hippocampus with several Glu analogues (Figure 2) [61,62,122,127,129,130,131]. Subsequently, neuronal damage and seizures may self-promote and regulate reciprocally in a positive feedback mechanism, wherein Glu is the neurotransmitter clearly implied. In addition, both GABAergic and glutamatergic cells may die, but interneurons seem to be more susceptible [61,129,130]. The effects of Glu depend on activation of several types of specific plasma membrane receptors (GluR), three of which are of the ionotropic (iGluR) type and are named by the selective agonists they are receptors for, namely, NMDA, AMPA (α-amino-3-hydroxy-5 methyl-4-isoxazole propionate) and kainate, which act as ligand-gated sodium/calcium channels; and eight of which are of the metabotropic type (mGluR), which are dependent on G proteins [119,120]. The iGluR antagonists block or reduce both neuronal death by Glu-mediated excitotoxicity and acute seizures generation but have poor efficacy in TLE epilepsy treatment [122]. Instead, GABA interacts with two general types of receptors, namely, one ionotropic known as GABA-A that acts as a ligand-gated chloride channel and the other as GABA-B, which is metabotropic and dependent on G proteins; [119] in this case, GABA-A receptor activation seems to be a common mechanism involved in the antiepileptic action of several drugs [122]. In addition, neuroplastic changes affect the neuronal signalling mediated by these neurotransmitters, as well as their transport, synthesis or degradation, such that neuronal inhibition is decreased and excitation is improved, resulting in the brain being more susceptible to seizures and to epileptogenesis [14,61,62,65,121,127,132,133].

During early development, both Glu and GABA exert neurotrophic effects, activating neuronal migration and axonal growth; moreover, GABA also controls these processes and promotes neurites outgrowth in the definition of dendritic arbors [125]. In later stages, long-lasting neuroplastic changes may be induced in the hippocampus by long-term potentiation (LTP) or long-term depression (LTD), which increase or decrease synaptic efficacy, respectively. Similar stimulation protocols or neural activity patterns can induce LTP in glutamatergic synapses and LTD in GABAergic, a condition that has been proposed as being determinant in both learning and epileptogenesis [14,61,62,122,127]. Establishment of LTP requires both pre- and post-synaptic depolarization, and GluRs activation, particularly of the NMDA receptors [14,61,62], whose composition, density and distribution are significantly modified in resected tissues of TLE patients and in samples obtained from several TLE experimental models [61,62].

Another plastic change that seems to be implicated in epileptogenesis involves to GABAergic neurotransmission, and it is related to the mechanisms that lead to GABA-mediated excitation through GABA-A receptor activation, which include changes in expression, viability or activity and cell distribution of two cation-chloride cotransporters (CCCs), namely, NKCC1 (chloride importer) and KCC2 (chloride exporter). When functional expression of KCC2 is higher than that of NKCC1, then GABA-A receptor activation promotes chloride entry and neuronal hyperpolarization, but when that relationship between these two CCCs is inverted, then GABA-A receptor activation depolarizes the neuron, and this condition has been related to seizures susceptibility and epileptogenesis in early development and in TLE [14,134,135].

Two important aspects should be noted. First, all statements mentioned above are based on the analysis of experimental acute and chronic epilepsy models, and of surgically TLE samples; second, not only have Glu and GABA been implicated in the plastic changes that mediate epileptogenesis, but also synaptic strength is highly susceptible to growth factors, other neurotransmitters, neuromodulators and hormones, which cannot be fully detailed in this review.

## 6. Neuronal and Glial Responses in the Hippocampus after Epileptic Seizures

Because neurons are not alone into the brain, one of the difficulties in the study of neuroplasticity and epilepsy, as in other neurological processes, was identifying all changes that occur in different cells in the orchestration of whatever process is being studied. However, in this section, we focused on the cells most closely related to synapses efficacy: neurons, astrocytes, and microglial cells. In addition to the modifications mentioned above, neurons that survive the degenerative process triggered by seizures modify the expression pattern and secretion of different neuropeptides and the density and distribution of their receptors, which may reduce the damage but also may reduce neuronal activation of GABA neurons or may increase Glu neurons activation [136], such as NPY and its receptors Y1 and Y2, whose expression is increased in both mossy fibres and GABAergic interneurons of the rat hippocampus in response to recurrent seizures. Similarly, the expression of those receptors also appears to be increased in surgically resected TLE samples, apparently reducing Glu release and neuronal excitation [113,137]. It should be mentioned that, even though interneurons are more vulnerable to the damage caused by seizures in comparison to dentate granular cells, dentate mossy cells are more sensitive than interneurons, and they respond to seizures extremely quickly and it is very complicated to identify the early changes produced in them [138]. Although some neuropeptides may reduce excitability, the progressive interneuron loss associated with seizures may promote a hyperexcitable state that complicates the control of them [139,140].

On the other hand, glial cells also respond to seizures and neuronal damage through a process known as “glial reactivity”, which comprises both morphological and biochemical changes. The morphological changes include cell proliferation and ramification of the cell processes of both astrocytes and microglial cells [141]; additionally, reactive astrocytes have shown a reduced capacity to maintain extracellular homeostasis at the level of ions, nutrients, and neurotransmitters, improving the hyperexcitable state [142], and even releasing Glu [126]. In addition, the BBB can be dramatically damaged, not only by changes in the functional expression of transport proteins expressed by the astrocyte but also because the astroglial feet retract and lose contact with endothelial cells [141]. Consequently, glial reactivity has a strong influence on neuronal functioning, particularly in the plastic changes related to epileptogenesis [143,144,145,146]. Furthermore, reactive glial cells increase the synthesis and secretion of chemokines and cytokines [147,148], which may improve astrocytes and microglial cells activation in a positive feedback signalling process. For example, pro-inflammatory cytokines, such as NF-κB and interleukin (IL)-1β, as well as its signalling receptor IL-1R1, are highly expressed by neurons and glial cells in TLE [149,150]. Recent studies carried out in different experimental models of epileptogenesis showed that epileptic seizures may induce glial reactivity [151,152,153,154] and increase pro-inflammatory cytokine levels, particularly in cerebral regions involved in the processes of both epileptogenesis and neuroplasticity [155,156,157]. These and other data allow the suggestion that seizures induce a strong inflammatory response that significantly modifies the functional interactions among microglial cells, astrocytes and neurons, which could be an important link between the two boarded processes here.

## 7. Neurogenic and Synaptogenic Responses to Epileptic Seizures

The integration of newborn neurons into the pre-existing circuitries of the adult hippocampus seems to have an important role in learning and memory in physiological conditions, but in pathological states this may induce aberrant synaptic reorganization neuroplasticity, contributing to the alterations [158,159]. Hippocampal neurogenesis has been considered an important factor in the pathophysiology of TLE over the last two decades [160,161,162,163,164,165,166]. Most of the neurogenesis occurs during early development, although certain brains regions maintain this neurogenic capacity throughout the lifespan, such as the DG wherein new cells appear to arise from the subgranular zone.

The neurogenic process that occurs after seizures has been demonstrated using experimental models and during other pathological events [167,168,169]. Weeks after the initial stimulus is presented, some neurons mature and are able to integrate into nearby neural circuitries [170,171], while many others may lose their way migrating towards the hilus and the CA3 area, where they constitute part of the pathophysiological changes. Electrophysiological studies have shown that the new cells have membrane properties similar to the mature cells located in the granular layer [171]. However, small differences have been observed, such as the presence of dendrites on both sides of the soma and the tendency to show epileptiform discharges spontaneously, with a frequency of 0.5 to 0.05 Hz.

In addition, the seizure type determines the neurogenic response, the amount of neuron newly produced, and aberrant migration; in particular, both excitotoxicity damage and neuronal denervation promote neuritogenesis [26]. As described above, in order for these modifications to be carried out, the expression of IEGs is necessary because they are responsible for the initiation of structural and functional changes through the regulation of secondary or late gene expression [172]. Both in the human hippocampus and in different experimental models, an intense re-innervation of the granular cells of the DG by mossy fibres has been observed, which contributes to the amplification of excitatory glutamatergic components, thus facilitating the unleashing of epileptiform seizures [160,173]. This cellular re-innervation could constitute a mechanism for the development and maintenance of epilepsy.

Another alteration in the hippocampus is the GABAergic cells loss and, consequently, the alteration in the mechanisms that regulate neuronal excitability, which are dependent on GABA as a predominant inhibitory neurotransmitter in adulthood. The disconnection of GABAergic interneurons from the hilus generates a disinhibition of the DG and the CA3 region, which also facilitates the discharge in the glutamatergic cells [40,174].

As was described earlier in this review, a large variety of plastic changes are generated in response to neuronal damage, especially after prolonged or repetitive seizures. Although, differential effects of the neurogenic role in epilepsy establishment are present and dependent on multiple factors, generally it is accepted that the outcome depends on synaptogenesis of the new neurons.

## 8. Conclusions

**Figure 3:**
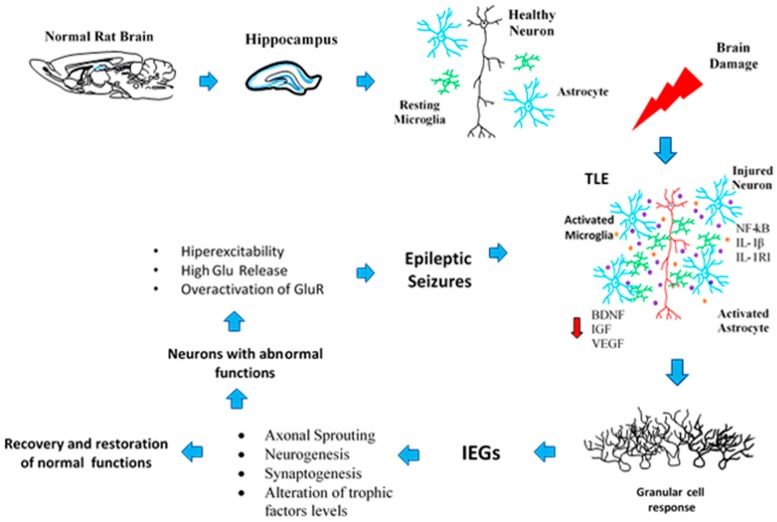
Schematic representation of the progressive events that lead to neuroplasticity and epileptogenic processes. First, the upper images refer to the undamaged hippocampus (blue colour) in a rat brain with most of the diverse cell populations (layers) represented (neuron: black colour; astrocytes: blue colour; and microglial cells: green colour). After non-lethal damage to the brain (red ray), reactive glial cells release pro-inflammatory chemokines and cytokines and modify neuronal activity (neuron: red colour; astrocytes: blue colour, and microglial cells: green colour; released pro-inflammatory molecules: purple colour; trophic factors levels are altered: red arrow). Then, through IEGs transcription, dentate granular cells respond to the damage through plastic changes that try to restore normal function but can also contribute to epileptogenesis, in a global process where in the mechanisms could affect on another.

The neuroplastic process has been considered both a cause and consequence of epilepsy, which represents more complexity than only the CNS restructuring. After seizure activity in the hippocampus not only does neuronal death occurs but also cell and molecular events that restructure and modify neuronal networks and synaptic communication occur; these modifications can reestablish normal functions or contribute to the development of neuronal illnesses, such as TLE. Growing evidence obtained through both experimental models and human brain samples has tried to explain some of the mechanisms involved in this type of illness, although many of them are contradictory. Specifically, in animal models, different factors must be considered, such as the mechanisms of damage induction, the animal species used, ages, genders, among others. Nevertheless, many groups of investigators continue to research the mechanisms implicated in neuroplasticity associated with epilepsy at different levels, especially in brain areas such as the hippocampus, which has a very important role in these processes (Figure 3).

Finally, one of the most important goals is to accomplish a clearly identification and understanding of the mechanisms and signalling pathways involved in neuronal death, plasticity, and epilepsy to identify new targets that may be used to develop therapeutic strategies to prevent or decrease the damages that lead to the establishment of neurological illnesses such as epilepsy.
